# Association of change on insulin-like growth factor (IGF)-i and IGF-binding protein 3 with genetic markers after a month of growth hormone (GH) therapy on Chinese children born with GH deficiency

**DOI:** 10.1186/1687-9856-2013-S1-P62

**Published:** 2013-10-03

**Authors:** Wei Wang, Shuixian Shen, Xiaoping Luo, Chunxiu Gong, Xuefan Gu, Yun Li, Minlian Minlian, Minlian Jin, Bin Wu

**Affiliations:** 1 Ruijin Hospital, Jiaotong University School Of Medicine, Shanghai, China; 2 Children's Hospital of Fudan University, Shanghai, China; 3 Tongji Hospital, Wuhan, China; 4 Beijing Children's Hospital, Beijing, China; 5 Xinhua Hospital, Shanghai, China; 6 Children's Hospital, Zhejiang University, Hangzhou, China; 7 First Affiliated Hospital, Sun Yat-sen University, Guangzhou, China; 8 Union Hospital, Wuhan, China; 9 Merck Serono China, Beijing, China

## Aims

To identify genetic markers associated with changes in IGF-I and IGFBP3 standard deviation score (SDS) after 1 month of r-hGH treatment in Chinese GHD children either born Appropriate for Gestational Age (AGA) or Small for Gestational Age (SGA).

## Methods

This phase IV open-label interventional study was performed on samples from 205 GHD children (175 subjects born AGA and 30 subjects SGA) of Chinese Han origin, recruited at 8 centers. All the subjects were given r-hGH for 4 weeks (0.033mg/kg/d).1536 SNPs were selected from 100 Candidate Genes involved in the GH-IGFI axes, growth plate and other short stature-related diseases. We genotyped Single nucleotide polymorphism (SNP) using Illumina GoldenGate™ Assays. Linear regression was used to identify single SNPs significantly associated with changes, from baseline to week 4, in serum IGF-I SDS and IGFBP3 SDS. Gestational age (GA) was also included in the model. The significance threshold was set as a corrected P-value<0.05. Multiple linear regressions with interaction effect were used to identify significantly associated genes with changes, from baseline to week 4, in serum IGF-I SDS and IGFBP3 SDS. The significance threshold was set as a corrected P-value<0.1.

## Results

(1) 6/19 SNPs which correspond to 4/17 genes were significantly associated with IGF-I SDS change/IGFBP3 SDS change separately from baseline to Week 4 in all samples (AGA+SGA) with a corrected P-value<0.05. Among them, 3/10 SNPs showed significant interaction effects, suggesting that the pattern of SNPs associated with IGF-I SDS change/IGFBP3 SDS change was different between two groups.

(2) 14/14 genes which significantly associated with IGF-I SDS change/IGFBP3 SDS change at Week 4 were identified in all samples (AGA+SGA). Among them, 6/12 genes showed significant interaction effects, suggesting that IGF-I SDS change/IGFBP3 SDS change have different effect on these genes expression between two groups. We also found that 5 genes in common associated with both IGF-I SDS change and IGFBP-3 SDS change.

## Conclusions

The results of our study demonstrate the genetic association between polymorphic variations of some candidate genes and serum IGF-I and IGFBP3 SDS changes after r-hGH therapy in children with GHD born SGA and AGA, suggesting that these genetic markers could be used into clinical practice in order to optimize efficacy, safety and cost of r-hGH therapy.

